# Using Immersive Virtual Reality to Examine How Visual and Tactile Cues Drive the Material-Weight Illusion

**DOI:** 10.3758/s13414-021-02414-x

**Published:** 2021-12-03

**Authors:** Caitlin Elisabeth Naylor, Michael J Proulx, Gavin Buckingham

**Affiliations:** 1 University of Bath, Bath, UK; 2 University of Exeter, Exeter, UK

**Keywords:** Perception, Multisensory, Vision, Touch, Virtual reality

## Abstract

The material-weight illusion (MWI) demonstrates how our past experience with material and weight can create expectations that influence the perceived heaviness of an object. Here we used mixed-reality to place touch and vision in conflict, to investigate whether the modality through which materials are presented to a lifter could influence the top-down perceptual processes driving the MWI. University students lifted equally-weighted polystyrene, cork and granite cubes whilst viewing computer-generated images of the cubes in virtual reality (VR). This allowed the visual and tactile material cues to be altered, whilst all other object properties were kept constant. Representation of the objects’ material in VR was manipulated to create four sensory conditions: visual-tactile matched, visual-tactile mismatched, visual differences only and tactile differences only. A robust MWI was induced across all sensory conditions, whereby the polystyrene object felt heavier than the granite object. The strength of the MWI differed across conditions, with tactile material cues having a stronger influence on perceived heaviness than visual material cues. We discuss how these results suggest a mechanism whereby multisensory integration directly impacts how top-down processes shape perception.

## Introduction

Perception is constructed through bottom-up processes that organize incoming sensory information along a feature-driven hierarchy and top-down processes that use prior knowledge, expectations, and predictions to interpret sensory input (Engel et al., 2001; Rauss & Pourtois, 2013). Perceptual illusions illustrate how these stimulus- and knowledge-driven processes interact to create unique perceptual experiences, rather than exact copies of the world (Coren et al., 2004; McMains & Kastner, 2011). For example, in the size-weight illusion (SWI), a smaller object feels heavier than a larger object, despite the objects having identical masses (Charpentier, 1891). The SWI can be partially explained by bottom-up processing of information relevant to the relationship between object size and mass, such as object density or rotational inertia, which is mistakenly interpreted as heaviness (Buckingham, 2014). Research also supports top-down explanations of the SWI which suggest that perceived heaviness reflects a contrast to the expected heaviness of the second lifted object in relation to the previous larger or smaller object (Buckingham, 2014). So, the SWI is at least partially driven by short-term expectations regarding the relationship between size and weight. For instance, if a large object is expected to be heavier than the previous smaller object, it will be lighter than expected and so feel lighter (Buckingham & Goodale, 2010). Accordingly, the SWI demonstrates how bottom-up and top-down processes are integrated to construct perception.

In some cases, perceptual illusions can be solely driven by long-term learnt associations and expectations. The material-weight illusion (MWI) describes a phenomenon whereby an object that appears to be made from a low-density material feels heavier than an object of equal weight, shape and size that appears to be made from a high-density material (Buckingham et al., 2009; Seashore, 1899). For example, when lifting two cubes with equal masses, the cube with a polystyrene surface is reported as feeling heavier than the cube with a metal surface, and this illusion persists across multiple lifts (Buckingham et al., 2009). The MWI is thought to be caused by a contrast between the expected and actual object weights, whereby the lower-density material feels heavier than expected in comparison to previous experiences with that material (Buckingham, 2014). These expectations are sufficient to drive the MWI in the absence of incoming visual information during the lift, as viewing the material of the cube prior to lifting the object whilst blindfolded induces an MWI (Buckingham et al., 2011). As such, the MWI demonstrates that perceived heaviness is not a direct measure of mass (or any other property of a stimulus) and can be driven solely by higher-level learnt associations between material and mass (Buckingham, 2014; Buckingham et al., 2011). This makes the MWI a useful tool for understanding how top-down processes can shape perceptual experiences.

Perception often relies on processing information received from multiple modalities and integrating these sensory cues to provide a unified percept. For instance, grasping and lifting an object requires visual information such as the location or size of the object, as well as tactile information received when the hand contacts the object, such as texture or hardness (Camponogara & Volcic, 2019). Multisensory integration can resolve perceptual ambiguities, enhance perceptual judgements and optimize action (Helbig & Ernst, 2007; Lalanne & Lorenceau, 2004; Talsma, 2015). However, the exact nature of this integration is dependent on the reliability and adaptive contribution of each modality to provide the most useful information for a specific situation, and so the perceptual benefit of multisensory integration is context dependent (Burke et al., 2006; Ernst & Banks, 2002).

The context-dependent relative contribution of sensory modalities has been demonstrated in the context of the MWI. Ellis & Lederman (1999) investigated the role of vision and touch in the MWI by asking participants to judge the heaviness of equally weighted objects with different surface materials. In the vision and haptics condition participants directly grasped and lifted the objects with full vision, in the haptic only condition participants directly grasped and lifted the object whilst blindfolded, and in the vision only condition participants lifted a board on which the object was placed with full vision. Visually-experienced material cues produced only a moderate strength MWI, whereas a much stronger MWI was experienced when the material cues were experienced with the fingertips (Ellis & Lederman, 1999). These results suggest that haptic information was both necessary and sufficient to produce a full-strength MWI, and thus haptics seemed to have a larger contribution than vision in this context. Such findings align with wider research showing that, although visual and haptic cues both contribute to identification and processing of material information, haptic cues are vital for material perception and thus understandably have a larger impact on the MWI (Baumgartner et al., 2013, 2015).

The influence of multisensory integration on bottom-up perceptual processes is reasonably well-studied, with several studies showing that the modality of incoming sensory information determines the exact content, organization and required processing of the sensory input (De Meo et al., 2015; Ernst & Banks, 2002; Lalanne & Lorenceau, 2004). However, little is known about whether the modality of incoming information can alter how top-down cues shape perceptual experience.

This study will investigate the interaction between the modality of incoming information and top-down perceptual processes in the context of the MWI. As the MWI is thought to be solely driven by higher-level expectations from previous knowledge of material-weight associations, it provides a way to focus on top-down perception alone. Unlike the Ellis and Lederman (1999) study, participants in this study used vision and touch simultaneously to experience the properties and judge the heaviness of lifted objects. Virtual reality was used to manipulate the congruency between visual and tactile material cues, whilst all other weight cues such as size and shape were kept constant. This mixed-reality paradigm provides a unique chance to manipulate the modality of material cues only and thus isolate the effect of modality on expectations derived from material cues. Consequently, this study will help understand how modality can influence perception at the point whereby expectations are derived from the environment to inform future percepts.

**Fig. 1:**
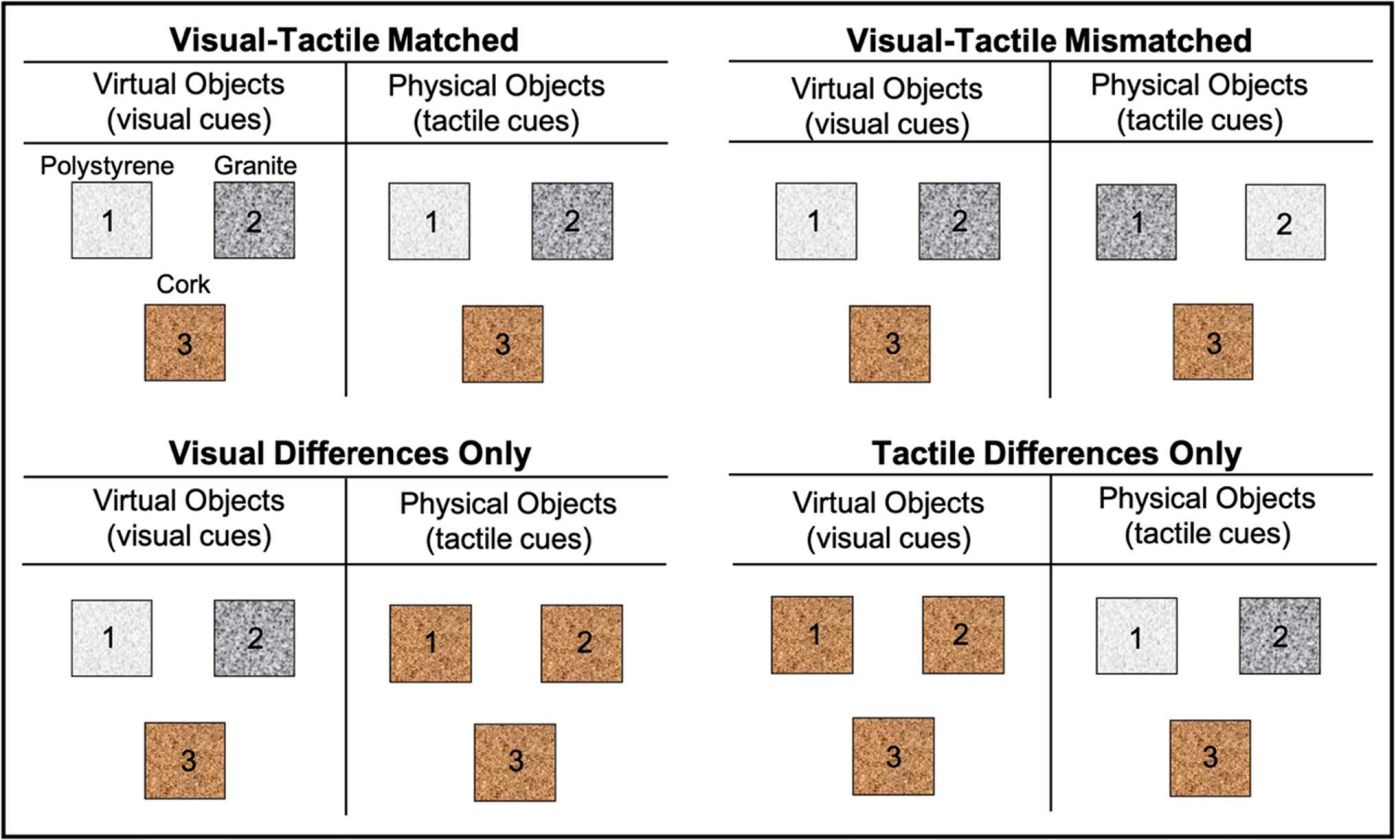
Physical and Virtual Cubes Used in Each Sensory Condition. *Note.* Diagram to illustrate which objects participants lifted in each sensory condition. Across all sensory conditions, participants always interacted with three different objects. The materials of these objects, both virtually and physically, differed depending on the sensory condition. The numbers represent which objects correspond to one another i.e. physical cube 1 was tracked and represented by virtual cube 1

The MWI was induced across four sensory conditions. Participants lifted equally-weighted physical objects made of different materials (polystyrene, granite or cork) whilst viewing computer-generated images of these objects moving in VR (Figure 1). In the visual-tactile matched condition, the physical object material matched the corresponding seen material in the VR environment. In the remaining three conditions, visual and tactile material cues were incongruent. In the visual-tactile mismatched condition, tactile material cues did not match the corresponding virtual material. In the tactile differences only condition, the physical objects were made of different materials, but the virtual objects were made of the same material, and so the material differences that drive the MWI were only available through tactile cues. In the visual differences only condition, the virtual objects were made of different materials but the physical objects were made of the same material, so material differences were only presented through visual cues.

These various configurations allowed us to examine how the modality through which an object’s material is experienced can impact perception of that object’s heaviness in the context of the MWI. Hypotheses were developed according to the discussed literature which demonstrated the benefits of multisensory integration for solving perceptual ambiguities (Lalanne & Lorenceau, 2004), and the stronger influence of touch over vision in the context of material perception (Baumgartner et al., 2015; Ellis & Lederman, 1999). Accordingly, we predicted that the MWI would be larger when differences between materials were presented through touch compared to vision (H1), and the MWI would be larger when visual and tactile material cues were congruent compared to when they are incongruent (H2).

### Method

#### Sample

To determine a target sample size, we used a recent meta-analysis to obtain the average effect size for an MWI, and calculated that this was 54% of the average effect size for the SWI (Saccone et al., 2019). We then calculated 54% of the smallest effect size found in Buckingham (2019) investigation of the SWI in different sensory conditions. This suggested an effect size of *dz* = .45 could be expected in the present study. A priori analysis showed that to detect this effect with .95 power in our planned follow-up t-tests, a sample of 55 would be required. However, due to the disruptions from COVID-19, the collected sample size was 29: this would give us a power of .76 to detect the estimated effect. One participant was excluded prior to data processing as they did not follow task instructions, leaving a final sample of 28, aged 18-24 (M = 18.8 years, SD = 1.2). There were 20 females and 8 males, 25 of whom were right-handed, and all were university students. Participants were recruited for convenience via the University of Exeter’s Psychology Research Participation Scheme, with ethical approval from the local ethics committee.

#### Design and Materials

**Fig. 2:**
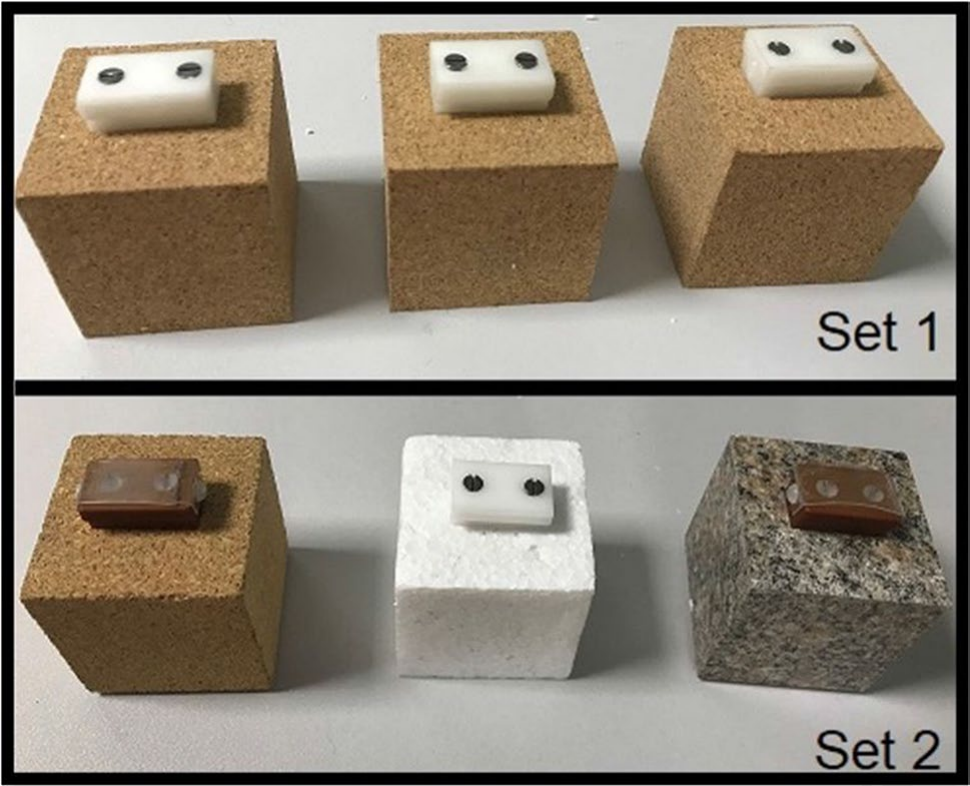
The Six Physical Cubes that Participants Lifted Across the Study.

All materials and data are available on the Open Science Framework (https://osf.io/7k548/). Participants lifted two sets of cubes of equal weight (123g) and equal size (5 X 5 X 5cm) (Figure 2). Set 1 consisted of three cubes with a cork surface material. Set 2 consisted of three cubes with surface materials of polystyrene (unaltered density 0.05g/cm^3^), cork (unaltered density 0.24g/cm^3^), and granite (unaltered density 2.67g/cm^3^). Each surface material was sealed around a hollow wooden box filled with lead shot and putty to adjust the weight to 123g, which was the average of the expected weight of a 5 X 5 X 5cm cube of each material based on the unaltered densities. Each cube had a handle attached to the top surface to allow a rigid body to be fixed on top. This was done in such a way that the objects appeared to be made completely from their surface material and did not appear to be tampered with (as in Arthur et al., 2019).

**Fig. 3:**
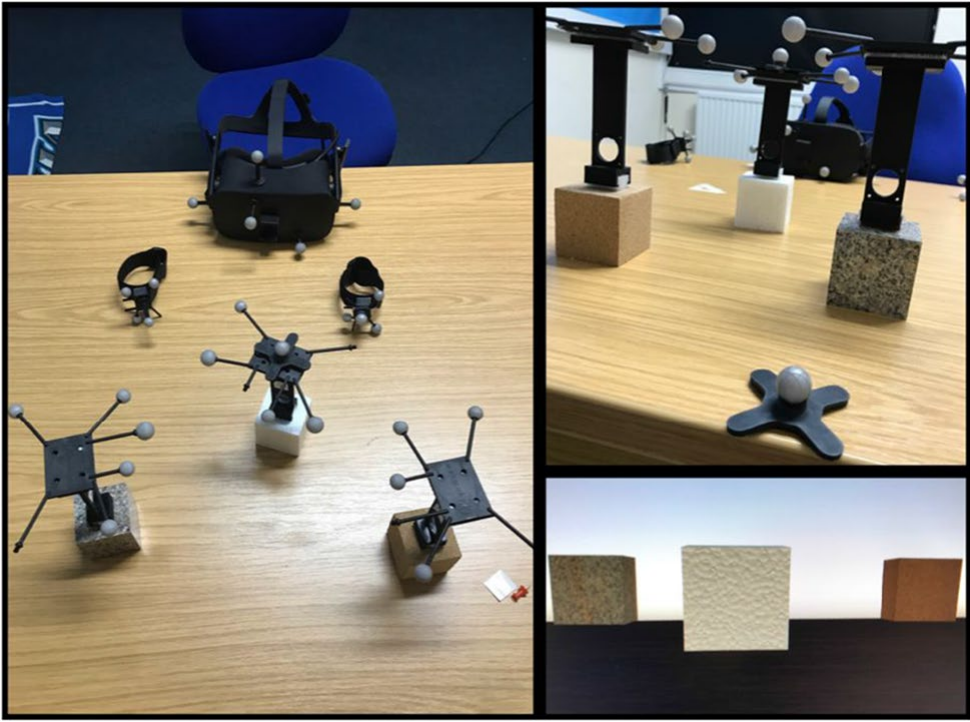
Tracking Physical Cubes in VR. *Note.* Participants wore the VR headset and wrist straps to interact with the objects in VR (right). Each physical cube had rigid bodies secured to the top (top right) to allow their movement to correspond to the movement of the replicated VR objects (bottom right)

Each set of cubes were replicated in an immersive VR game environment designed using Unity game engine, displaying a simplified version of the testing lab. Unique 5/6-marker-configuration rigid bodies were used to track the movement and position of the physical cubes, the Oculus headset, the table surface, and wrist straps, tracked by an eight-camera Optitrack Flex 13 (NaturalPoint Inc. Corvallis, OR) motion capture system. This allowed participants to interact with the physical objects whilst viewing them in VR. Participants wore an Oculus Rift CV1 head mounted display (HMD) (Oculus VR, Irvine, CA) which was defined as the scene viewing camera, allowing participants to dynamically view the scene. The positions of the HMD, table and wrist straps were tracked in relative positions to one another, with the table having roughly the same proportions as the real table surface and the wrist markers rendered as small orange spheres. High-quality images of the materials were taken from the internet to create the materials for the virtual objects, to match the physical objects. These objects were positioned according to the rigid body positions in such a way that the bottom of the VR object appeared to rest on the virtual table surface when the physical objects were placed on the table, allowing for smooth reaching and grasping. The virtual cubes were mapped on to the physical cubes through trial and error: rough mapping was implemented and then members of the lab viewed the cubes in VR and interacted with the physical cubes and provided feedback until the size and appearance of the VR cubes appeared congruent to the user. The physical experimental set up and virtual cubes are shown in Figure 3, and the above design is similar to that used by Buckingham (2019).

We used a repeated-measures design to investigate perceived heaviness across four conditions, illustrated in Fig. 1. In the visual-tactile matched condition, participants lifted the physical cubes from Set 2, which matched the cubes in VR. In the visual-tactile mismatched condition, participants physically lifted and viewed the cubes from Set 2; however, the visual and tactile materials were not matched, such that the physical polystyrene looked like granite in VR and vice versa (as a control, the physical cork looked like cork in VR). In the visual-differences only condition, participants lifted the cubes from Set 1 but viewed the cubes from Set 2 in VR, so that differences between materials were only presented visually. Contrastingly, in the tactile differences only condition, participants lifted the cubes in Set 2 but viewed the cubes in Set 1 in VR so that differences between materials were only presented through touch. This experimental design meant that the mass, size and shape of the cubes were always the same and that participants always experienced the properties of the cube they were lifting through both vision and touch: the only differences between the conditions was how the material information was presented.

#### Procedure

Participants were informed that the purpose of the study was to understand how materials were perceived in VR. Participants were told that they would lift cubes made of different materials and judge how heavy they felt when viewing them in VR. Upon receiving detailed study instructions, participants attached the wrist straps and placed the HMD over their head, which was adjusted until comfortable. Participants viewed the three cork objects in the headset and were asked to reach out and lift the object closest to them – this allowed participants to practice a trial and adjust to reaching for the objects in VR.

Once participants confirmed they were comfortable with the task, we measured conscious expectations of heaviness for each material. The objects in Set 2 were displayed in VR and, without touching the physical cubes, participants were asked to judge how heavy they expected each object to be. Participants were asked ‘how heavy do you expect the polystyrene/cork/granite cube to feel’: these three questions were only asked once at the beginning of the study. Participants were asked to give a numerical rating on a scale of their own choosing (i.e., an absolute magnitude estimation) to rate how heavy they expected the objects to feel (Zwislocki & Goodman, 1980). Participants were told they could use any numbers that made sense to them (e.g., negatives, decimals, 10s, 100s etc.) and that they should use a consistent rating scale across the conditions. This is a standard measure of perceived heaviness used in weight illusion studies and is useful for capturing the subjective judgement of each participant whilst still providing a quantifiable measure that can be standardized and used in later analysis (Buckingham et al., 2011; Naylor et al., 2020).

Next, participants completed the main experimental trials. Each condition consisted of 24 lifts (8 of each material), with cubes presented in one of three random orders across participants. The order in which participants experienced the 4 conditions was counterbalanced, and participants had a 3-minute break between conditions during which time they removed the HMD. On each lift, participants were told to close their eyes (whilst wearing the HMD) so that the experimenter could position the 3 cubes centrally in front of them, in a triangular formation. Participants were then asked to open their eyes and lift the cube closest to them a short distance off the table, for a short period of time (usual hold time was 1-2 seconds - the experimenter ensured lift time was consistent within-participants). No specific instructions were given regarding how they should grasp the cube; however, all participants grasped the cube by the left/right sides of the cube to ensure they did not knock the rigid bodies affixed to the top. Participants then rated how heavy the cube felt using their chosen scale and this process was repeated for the next lift.

### Data treatment

Participants’ heaviness ratings were transformed to z-scores within subject, using the mean and the standard deviation of individuals’ values. The material labels given to the data always referred to the physical materials (i.e. the scores for polystyrene referred to the physical polystyrene), except in the visual condition were material differences were only present visually. We then calculated each participant’s average z-score rating given to each cube in each condition. This yielded 12 groups of mean averages – averages for the three materials across the four conditions – which were examined in the ANOVA.

Three average z-score ratings were identified as outliers (tactile-granite for participant 11; matched-polystyrene and mismatched-polystyrene for participant 12). These scores exceeded the upper or lower bounds by 1.5 times the interquartile range when compared to the other average scores given to the same material in the same condition (Tukey’s rule). As these outliers showed extreme opposite trends to that expected in a MWI (i.e. the polystyrene was rated extremely light or the granite extremely heavy), they were removed. Outliers were removed using pairwise deletion to minimize data loss, meaning that participant 11 had one missing data point and participant 12 had two. After outliers were removed, we had a final sample of 28 participants for nine groups of averages and 27 for the three groups in which the outliers were removed. This final data set was normally distributed, with non-significant Shaprio Wilks tests for all 12 groups of averages and Q-Q plots showed no visual distribution abnormalities. Lastly, due to the unforeseen interruptions to testing, we calculated the sensitivity of our tests, and found that using a two-tailed test with alpha of .05 and power .95, we would be able to detect an effect of *dz* = 0.72 with a critical *t* = 2.06.

## Results

**Fig. 4:**
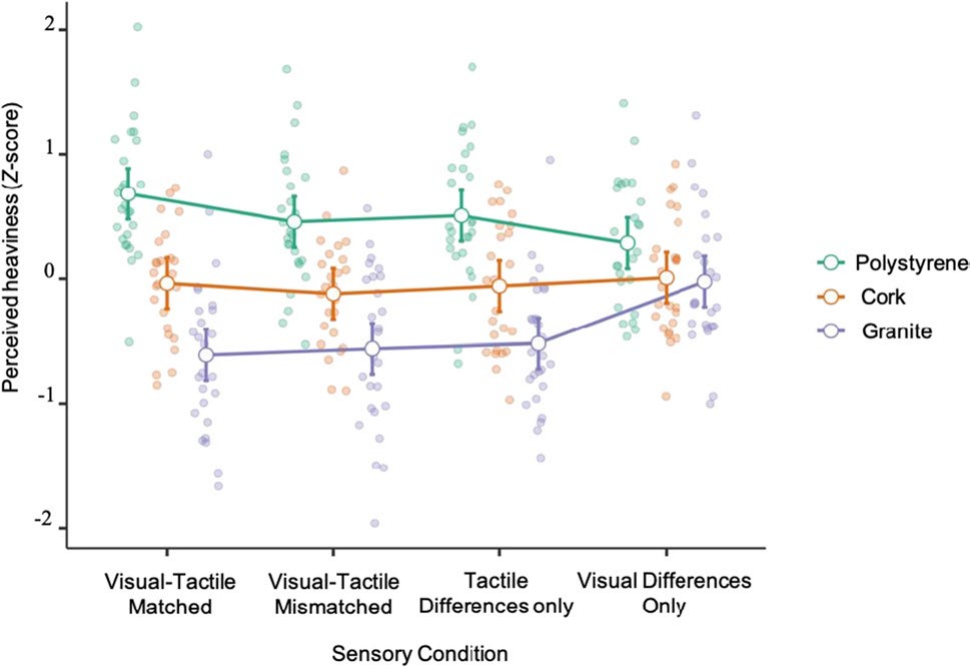
Interaction Effects of Material and Sensory Condition on Perceived Heaviness. *Note.* The white circles represent the group mean z-score, and the individual points represent each participant’s mean z-score. The error bars represent 95% confidence intervals

Initial exploration of the data showed that in all sensory conditions, polystyrene was perceived as the heaviest object and granite as the lightest, showing the expected trend for a typical MWI (mean z-scores shown in Fig. 4). Importantly, the pre-lift ratings complimented these trends. All participants expected the polystyrene object to be lighter than granite, although the expectations about the cork object were more variable. When averaging participants’ raw pre-lift ratings, the typical trend emerged with polystyrene expected to be the lightest (M = 3.35), followed by cork (M = 5.54) and then granite (M = 9.50).

### Omnibus analysis

We then analyzed this data using a 3x4 (material x sensory condition) repeated-measures ANOVA to investigate how material and sensory conditions influenced perceived heaviness. Mauchley’s test indicated no violations of sphericity for the sensory condition (W = .94, *p* = .92), materials (W = .94, *p* = .46) and interactions (W = .26, *p* = .06). There was no main effect of sensory condition (F(3) = .58, *p* = .63), which was expected as the differences between the sensory conditions were only present across material information. In contrast, we found that differences between cube material had an extremely large effect on perceived heaviness, independent of sensory condition (F(2) = 68.59, *p* < .001, η_p_^2^ = .73), indicating that a MWI was induced across the study. Importantly, we also found there were substantial interaction effects (F(6) = 9.73, *p* < .001, η_p_^2^ = .28), suggesting that the modality through which material information was presented influenced the resulting MWI (Fig. 4). However, these effects do not reveal exactly how the MWIs were different in each sensory condition, and thus further analysis was required.

The main effect in the ANOVA showed that an MWI was induced when controlling for sensory conditions, however as our hypothesis focus on the differences in the MWI between each sensory condition, we needed to confirm that a significant MWI was induced in each sensory condition. To do this, we calculated the magnitude of the MWI in each condition by subtracting the granite z-score rating from the polystyrene z-score rating of the same trial (i.e. polystyrene lift 1 – granite lift 1 etc.). The outliers that were already identified did not undergo this process (i.e. participant 11 did not have a magnitude score for the tactile condition, as the granite score was an outlier), so the magnitude scores were calculated from the same data used in the ANOVA. We then averaged these scores within participants so that each participant had four magnitude scores, one for each sensory condition. This produced a single measure to represent the strength of the MWI, facilitating our subsequent analysis.

**Fig. 5:**
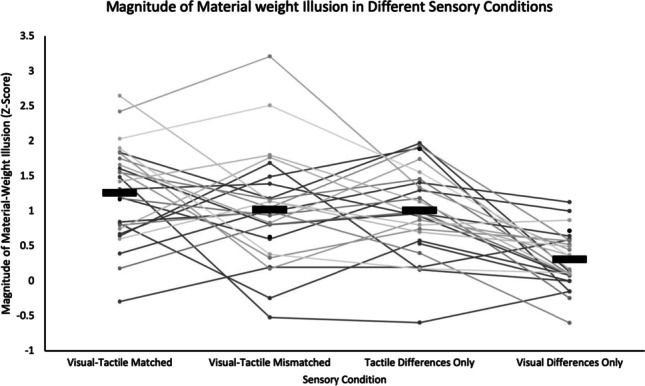
Magnitude of the Material-Weight Illusion in Each Sensory Condition. *Note.* The black bar represents the mean magnitude. The individual dots represent each participant's average magnitude (each participant’s data is joined by the lines).

Next, we calculated the mean MWI magnitude for each sensory condition and confirmed that participants experienced an MWI in all conditions and one sample t-tests confirmed that the MWI magnitude in each condition was significantly different than 0 (Fig. 5). The visual-tactile matched condition produced the largest MWI (M = 1.3, SD = .68), shown by the extremely large effect size (t(26) = 9.59, *p* < .001, CI = .99-1.53, *dz* = 1.85). The visual-tactile mismatched (M = 1.02, SD = .78) and tactile differences conditions (M = 1.01, SD = .63) produced similar MWIs, with the tactile differences condition having a slightly stronger illusion show by the larger effect size (t(26) = 8.25, *p* < .001, CI = .76-1.26, *dz* = 1.59) than the visual-tactile mismatched condition (t(26) = 6.78, *p* < .001, CI = .71-1.33, *dz* =1.31). Finally, the weakest MWI emerged in the visual differences condition (M = .31, SD = .40) and despite having a comparatively smaller effect size, the MWI was still significant (t(27) = 4.11, *p* < .001, CI = .15-.46, *dz* = 0.78). Altogether, this confirms that a robust MWI was present in each individual sensory condition, and thus it is appropriate to perform further analysis to investigate the differences between each MWI.

#### Investigating specific hypotheses

The interaction effects of the ANOVA showed that sensory modality impacted the strength of the MWI, however did not reveal the exact nature of this impact. Consequently, to answer our specific hypotheses regarding the strength of the illusion in each condition, we used six planned paired-samples t-tests to compare the MWIs in each sensory condition. To account for multiple comparisons, p-values were considered significant if they met a Bonferroni-adjusted threshold of p = .008. When differences between materials were presented through vision only, the resulting MWI was significantly weaker than the MWIs in the visual-tactile matched (*t*(26) = 6.24, *p* < .001, 90% CI [*0.63-1.25*], *dz* = 1.20), visual-tactile mismatched (*t*(26) = 4.12, *p* < .001, 90% CI [*0.35-1.05*], *dz* = 0.79) and tactile differences only conditions (*t*(26) = 5.65, *p* < .001, 90% CI [*0.45-0.97*], *dz* = 1.09). The substantial effect sizes show that sensory presentation of material had a profound influence on differences in perceived heaviness, whereby visual presentation significantly reduced the strength of the MWI. Contrastingly, there were no significant differences identified between the matched and mismatched (*t*(26) = 1.41, *p* = .17, 90% CI [*-0.11-0.60*], *dz* = 0.27), matched and tactile-only (*t*(25) = 1.67, *p* = .11, 90% CI [*-0.06-0.60*], *dz* = 0.33) or mismatched and tactile-only conditions (*t*(25) = .051, *p* = .96, 90% CI [*-0.31-0.32*], *dz* = 0.01). Altogether, these results highlight exactly which sensory conditions produce different MWIs and thus demonstrate how modality through which materials are experienced can influence the perceived heaviness of an object.

## Discussion

In this experiment, we sought to examine how the modality through which an object’s material is experienced can impact perception of that object’s heaviness. A material-weight illusion (MWI) was induced by presenting participants with different materials using congruent and incongruent tactile and visual cues. As predicted, an MWI was induced using visual and tactile cues alone, as well as when combining these cues, showing that both tactile and visual material cues activate expectations of object weight that drive illusory heaviness differences. Furthermore, the MWI was stronger when differences between materials were presented through touch compared to vision, supporting hypothesis 1. The MWI induced with matched visual and tactile cues was stronger than the visual differences only condition, however, was no different to the other two conditions. This shows that congruent visual and tactile material differences did not consistently produce a stronger MWI than when incongruent visual and tactile cues were used, providing only partial support for hypothesis 2. Altogether, these results demonstrate that manipulating the modality through which materials are presented can affect the magnitude of the MWI, even when other weight cues remain constant.

Comparison of the MWI across the sensory conditions suggests that higher-level expectations derived from tactile material cues exert a more substantial influence on heaviness perception, compared to visual material cues. Specifically, when expectations of object weight were induced from visual material cues, a weaker MWI occurred than when the same expectations were trigged by tactile or matched visual-tactile material cues. From this it can be assumed that tactile material cues had a larger influence on perceptual judgements of heaviness than equivalent visual material cues. The similarity between the tactile and matched visual-tactile MWIs support this notion, suggesting that when tactile cues are available, visual material information does not have a significant additive contribution. These observations compliment Ellis & Lederman's (1999) previous findings that haptic material cues were necessary and sufficient alone for a full strength MWI. An interpretation that tactile cues dominate visual cues in the MWI fits with wider research which demonstrates that tactile cues are vital for material perception (Baumgartner et al., 2013) and that information about material can be extracted more quickly from tactile cues, increasing perceptual efficiency (Klatzky et al., 1993). A tactile dominance effect could be explained by optimal integration theories of multisensory perception, which would suggest tactile material information was more useful and/or reliable to the perceptual task than the visual cues, and so had a larger relative contribution to the final perceptual judgement of heaviness (Ernst & Banks, 2002; Lalanne & Lorenceau, 2004). From this, it can be suggested that the higher-level expectations derived from tactile material cues had a more substantial impact on shaping heaviness perception than when the same material information was presented visually.

The enhanced contribution of expectations derived from tactile cues in the MWI is more dramatically evident in the mismatched visual-tactile condition. When both visual and tactile cues suggested material differences that were incongruent with one another, a tactile MWI (physical polystyrene object judged to be heavier than physical granite) occurred that was no different to that found when no visual material differences were present. In other words, when visual material cues activate conflicting expectations of heaviness differences (as opposed to none or matching), they do not dampen the influence of expectations derived from tactile cues. A potential explanation for this is that when sensory information is combined, the information from one sense can be used to judge the reliability of the other (Atkins et al., 2001). So, the reliability of the visual information may have been judged to be poor because it directly conflicted with information from the more contextually-relevant tactile information (Gibo et al., 2017). Importantly, this condition provides further evidence for an interaction between modality and higher-level expectations in heaviness perception. Furthermore, this study extends beyond the context of the MWI by finding evidence of a modality-specific modulation on top-down perceptual processes. By only altering the modality of the material information which drives the illusion, this study demonstrates how the modality of incoming sensory information can determine how prior knowledge and expectations shape perception.

Whilst it is clear that multisensory integration influences top-down perceptual processes, the presence of an (albeit weaker) MWI in the visual condition shows that tactile cues do not always dominate visual cues. The dominance of visual cues in this condition may be due to material differences (presented visually) having a stronger influence than similarities between materials (presented tactilely). Research in habituation and sensation shows that perceptual processing is more sensitive to changes between objects, which may explain the larger contribution of visual material differences (Gati & Ben-Shakhar, 1990; Horstmann & Herwig, 2015). This mirrors previous research that multisensory integration, that is the relative contribution of vision and touch, depends on the specific information conveyed by each modality. Nevertheless, the visual MWI was significantly weaker than all others, showing that tactile cues (which suggested no material differences, and was thus associated with expectations of equal object weight) dampened the influence of visually-derived expectations. Consequently, the observations across all four conditions illustrate that the modality of incoming information can modulate how higher-level knowledge shapes perception.

Although this study reveals how modality can influence top-down perceptual pathways, the results do not indicate exactly when this modulation occurs. For example, it is possible that the reliance on tactile cues meant that expectations were only derived from tactile cues, or conversely expectations may have been derived from visual and tactile material information but the tactilely-derived expectations outweighed the visual. This study was a useful first step to understand the important role of multisensory integration in top-down perceptual pathways, but further investigations will be needed to develop understanding of the mechanistic role of modality.

In addition, this study is limited by using VR to present the visual material cues. Virtual visual cues have a lower resolution and issues with vergence/accommodation conflict which may have reduced the reliability of the visual cues. Whilst it is unlikely that this accounts for all the findings because results align with previous work, future use of VR for perception research should aim to increase the ecological validity and validate representation of visual cues in VR. For example, adding indirect visual cues such as indentations on materials or sight of fingers grasping the object could provide a richer visual experience. Consequently, this partially limits the generalizability of our results outside of the VR environment, whereby the extreme dominance of tactile cues may not be replicated when using naturally-occurring visual cues. On the other hand, VR provided a unique opportunity to isolate the effects of individual modalities and was the first study, to the author’s knowledge, to replicate the MWI in VR. As VR continues to develop, perceptual phenomena can be explored from new angles and existing observations can be validated further.

In conclusion, this study demonstrates that whilst both visual and tactile cues contribute to the MWI, when the expectations that drive the MWI are derived from tactile material cues, the resulting MWI is stronger than when the same information is presented visually. Consequently, these findings have highlighted how the modality through which material cues are presented influences how expectations about material weight drive heaviness perception. More broadly, by manipulating the congruency of visual and tactile cues and thus retaining a multisensory context throughout the study, we have identified an interaction between modality and higher-level expectations whereby multisensory integration directly influences top-down pathways that shape perception.

## Declarations

### Statement of Significance

This study furthers our understanding of how multisensory integration actively shapes our perception of the environment. We have provided a better understanding of the material-weight illusion, showing that when expectations about weight are derived from tactile material cues, the resulting illusion is stronger than when visual cues drive the illusion. Importantly, this shows an interaction between higher-level expectations that drive heaviness perception and multisensory integration, appreciating the rich context in which perception is constructed. Future research should appreciate the mechanistic role of modality and consider the influence on top-down perceptual processes.
